# Supplementary material for: Automated cardiovascular magnetic resonance image analysis with fully convolutional networks
Source: J Cardiovasc Magn Reson. 2018 Sep 14;20:65. doi: 10.1186/s12968-018-0471-x (PMC6138894; doi:10.1186/s12968-018-0471-x)

An in-house software generates segmentation images, which shows automated segmentation, manual segmentation and the difference map between the two segmentations to facilitate image analysts in visual qualitative assessment.

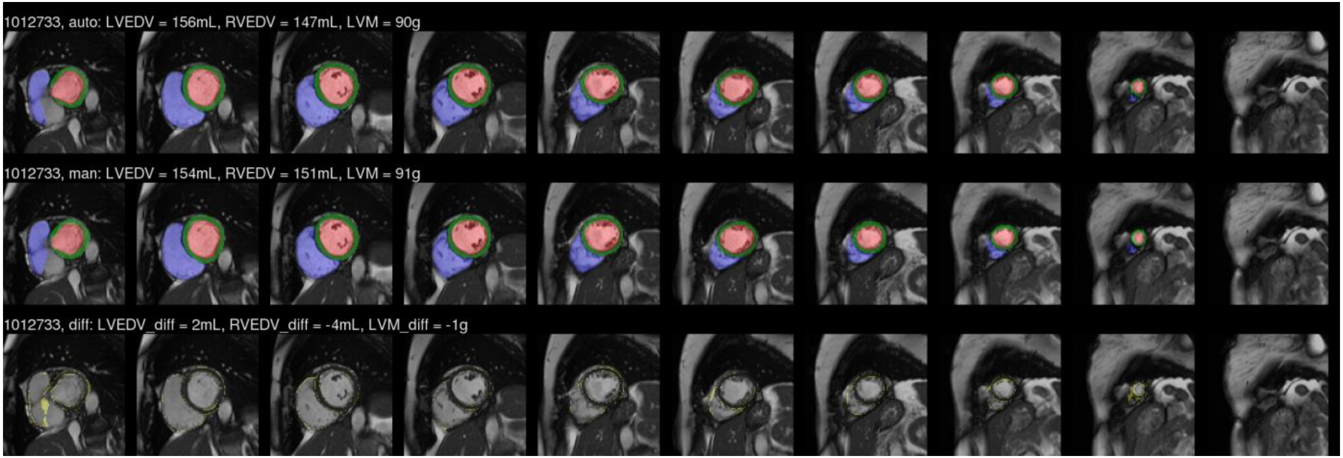

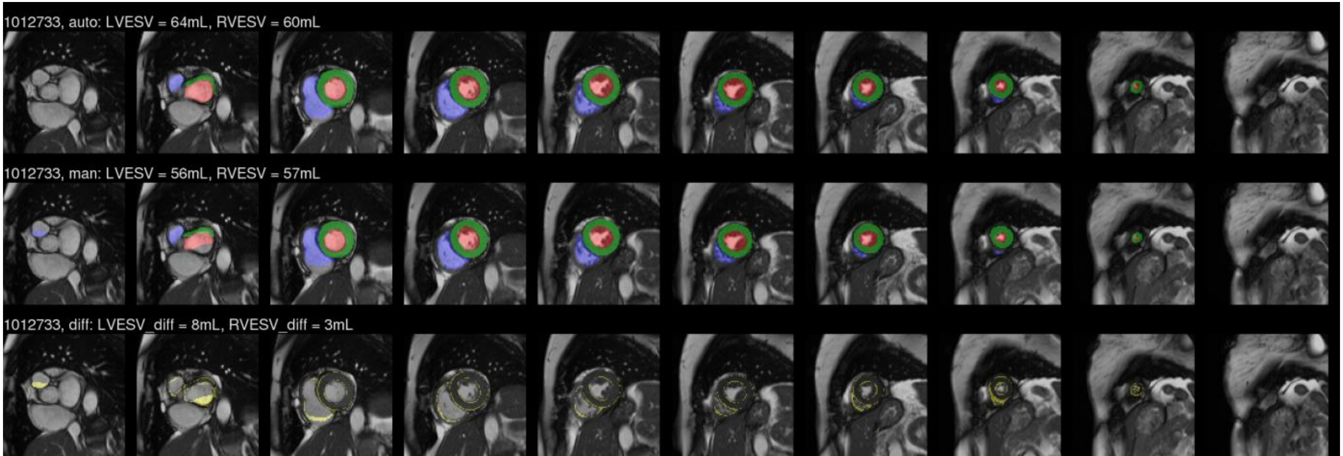

Supplement: Supplementary file 1 — Image demonstrating visual assessment and comparison between automated segmentation and manual segmentation. (PDF 210 kb) [file 12968_2018_471_MOESM1_ESM.pdf]
